# Sap flow of *Salix psammophila* and its principal influencing factors at different slope positions in the Mu Us desert

**DOI:** 10.1371/journal.pone.0225653

**Published:** 2019-12-05

**Authors:** Zhiyong Pei, Shaorong Hao, Guohui Pang, Kai Wang, Tiejun Liu

**Affiliations:** 1 College of Energy and Transportation Engineering of Inner Mongolia Agricultural University, Hohhot, Inner Mongolia Autonomous Region, China; 2 Institute of Water Resources for Pastoral Area, Ministry of Water Resources, Hohhot, China; CAS, CHINA

## Abstract

The changes in sap flow of *Salix psammophila* growing on a gentle slope (lower slope, P1), a middle slope (P2), and an upper slope (P3), and the response of sap flow to meteorological factors at the different slope positions were studied using the continuous and synchronized observations, the instrument were wrapped stem flowmeter EMS 62 sap-flow heat-balance-based system and the LSI-LASTEM automatic weather station. The results revealed that the soil moisture content was the highest and the growth conditions of *Salix psammophila* were the best at P1, followed by P2. At P3, however, although good apical dominance was observed, the proportion of dead branches was the highest. Furthermore, the daily variation patterns of sap flow on the three slopes presented as multi-peak bell-shaped curves. The daily accumulation changes in sap flow showed a trend of P1 > P3 > P2, and within the same diameter range, the sap flow at P1 was significantly different from that at P2 and P3, whereas the sap flow at P2 and P3 did not vary significantly. All the three slopes showed a significant and positive correlation with photosynthetically active radiation, atmospheric temperature, and vapor pressure difference, and a significant and negative correlation with relative humidity; however, the degrees of correlation varied slightly. The stepwise regression analysis showed that, at different slopes, different variables were selected for different branch diameters, but photosynthetically active radiation and atmospheric temperature played dominant roles on all slopes. This study reveals the sap flow pattern of *Salix psammophila* on different slopes and its response mechanism to meteorological factors, which was essential for understanding the restoration ability, physiological adaptability, and ecosystem stability of *Salix psammophila* communities.

## Introduction

The Mu Us Desert is one of the four major sandy areas in China, with a typical fragile ecosystem of arid and semi-arid lands. The area is one of the key areas of wind-blown sand control in China, because of its single tree species and sparse biological population [1,2]. *Salix psammophila* (also known as desert willow) has many characteristics, such as fast growth and a strong germination ability, tolerance to drought, wind-erosion, and sand burial, and good sand-fixing and water-conservation qualities [3–5], which have led to its use in sand dune stabilization and water conservation in this region. The large-scale planting of *Salix psammophila* has significantly improved the overall quality and effectiveness of desertification prevention in this region.

However, artificial afforestation of sandy areas has led to excessive water consumption, mainly because of an overly dense plant distribution or a large water consumption by vegetation. Moreover, this region has low annual precipitation, which is characterized by high interannual variations and uneven distribution; for many years, the maximum mean annual precipitation and mean annual evaporation rate here have been 300 mm and 2000 mm, respectively. As water loss from surface evaporation exceeds the amount of water replenished by rainfall and because *Salix psammophila* plants consume large amounts of water through transpiration, the groundwater supply has become seriously depleted, creating a serious imbalance between the water supply of sandy soils and the water demand of plants, which has led to the decline and death of *Salix psammophila* populations. Therefore, water supply has become the major factor limiting the growth of *Salix psammophila* [6,7]. Studies have shown that only 1%–5% of the water that is transported from roots to leaves is metabolized by a plant, whereas the remaining 95%–99% of the water is dissipated into the atmosphere [8]. Therefore, a comprehensive study of transpiration in *Salix psammophila* plants growing in this region is of considerable importance, because it can guide rational planting of *Salix psammophila*, given the limited water resources, and can be used to formulate scientifically sound management measures.

Transpiration reflects the growth condition of vegetation; the process and rate of transpiration of individual plant can be accurately estimated through sap flow measurement [9]. Some studies on sap flow in psammophytes have already been published [10–13]. More specifically, Hong Guangyu et al. [14] analyzed the sap flow characteristics of *Hedysarum fruticosum* var. *mongolicum* under sunny conditions and after rainfall. Xu Dandan et al. [15] found that the sap flow densities of *Salix matsudana* and *Populus simonii* had the best correlation with net radiation. Fan Wenhui [16] studied the differences in sap flow of *Hedysarum scoparium*, *Pinus Sylvestris var*. *Mongolica*, and *Populus bolleana Lauche* under different atmospheric conditions. Xin Zhiming et al. [17] found that, during the fruiting season of *Hippophae rhamnoides*, air relative humidity was one of the major meteorological factors affecting sap flow. Wang Qiangmin et al. [18] found that the sap flow velocity of *Salix psammophila* was affected significantly by an atmospheric vapor pressure deficit. Pei Zhiyong et al. [19] studied the characteristics of the sap flow of *Salix psammophila* branches under different meteorological conditions. The topography of the Mu Us Desert is dominated by gently sloped sand plains and sand dunes. However, most current studies have concentrated on sap flow in psammophytes growing on desert plains, and the dynamic changes in sap flow owing to different slope positions have been neglected. In arid and semi-arid regions, where environmental factors are complex and sap flow rates are sensitive to environmental changes [20], it is important to investigate the effect of different slope positions and dominant environmental factors on sap flow to accurately estimate water consumption in sandy regions.

In this study, we studied the response of *Salix psammophila* at different slope positions to the environmental factors of the Mu Us Desert. By analyzing how different slope positions influence the relationship between sap flow characteristics of *Salix psammophila* and meteorological factors, we obtained a more accurate and comprehensive variation pattern of sap flow of *Salix psammophila* plants. This study provides a theoretical basis for elucidating the mechanism of water consumption through transpiration of psammophytes. And will give practical guidance for plant restoration and shrub plantation management in sandy areas.

## Materials and methods

### Ethics statement

The study site is owned by Ordos city, Inner Mongolia autonomous region. The field work did not involve any endangered or protected species, and did not involve destructive sampling. Therefore, no specific permits were required for the described study.

### Study area

The selected study area was the Wushenqi Sand Control Station (38°52′N, 109°12′E), which is located in the Mu Us Desert hinterland. The total area is 309 km^2^, with a mobile dune area of 152 km^2^. The climate here is of the temperate arid and semi-arid type, with a mean annual temperature of 7.5°C, an effective accumulated temperature of 2800°C, an annual temperature range of 30°C, a mean daily temperature difference of 13.3°C, and an annual sunshine duration of 2500 h. The annual mean wind speed is 3.4 m/s, and the prevailing wind direction is NW throughout the year. The mean annual precipitation in this region is 300 mm, with precipitation mostly occurring in July and August. The mean annual evaporation rate is 2000 mm, with an Aridity Index of 1.9. The dominant soil type is chestnut soil with a small amount of brown soil. Shrub species include *Salix psammophila*, *Artemisia sphaerocephala*, *Caragana korshinskii*, *Hedysarum fruticosum var*. *mongolicum*, and *Hippophae rhamnoids* [21].

### Experiment design

**Fig 1:**
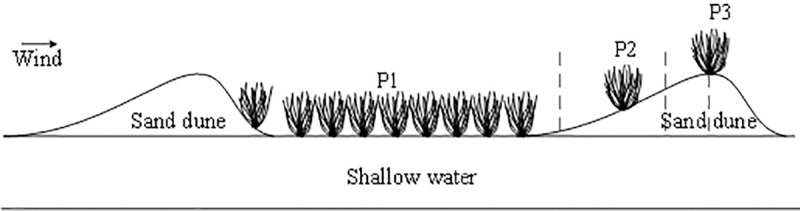
Schematic diagram of plot terrain.

First, a typical slope (a horizontal length of 350 m, a slope angle of 32°, and a vertical drop of 100 m) of the *Salix psammophila* plantation running in the northwest direction (main wind direction, 75° NW) was selected in the study area. The selected slope was then divided into three sections, namely, the gentle plain section (P1), the middle slope section (P2), and the upper slope section (P3) and a sample plot of 20 m × 20 m was set up at each of these sections. The age of *Salix psammophila* in the sample plots ranged from 3 to 5 year. The spacing of plants and plant rows in P1 was 1 m × 3 m, whereas fewer plants with a scattered distribution were found in P2 and P3. A meshed fence was used to enclose the plots, to prevent livestock grazing nearby from entering the plots to forage and from destroying the sensors. Fig 1 shows a schematic diagram of the selected slope terrain. The experiment was performed in July 2019 ranged from the 10th to the 15th.

### Determination of sap flow rate

In each plot, three healthy *Salix psammophila* specimens were selected and their branches were categorized into three diameter ranges [19]: 4–8 mm (D1), 8–12 mm (D2), and 12–16 mm (D3). Next, some branches from each diameter range were selected as test specimens. The branch diameters were measured at a distance of 45 cm from the ground surface using a Vernier caliper, ensuring that the measurement error was within 0.5 mm, and recorded the date. Sap flow measurements were carried out using a strap-on EMS 62 sap-flow system (Chech Republic Dynamax) [22,23] installed on the tested branches at a height of 45 cm above the ground surface. The sap-flow system based on principle of heat balance, which was used to determinate the flow rate of crops, seedlings and twigs with relatively, and will protect the branches from damage. Sample data were collected every 30 min using a Campbell Scientific CR-1000 data logger. Sap flow sensors were installed using the same method as described by Yue Guangyang et al. [24], whereas the principles and steps of the sap flow measurement can be found in Pan Tianhao [25] and Wei Xinguang et al. [26].

As the water level in the P1 sample plot was relatively high and the main suction root system of *Salix psammophila* can reach a depth of about 30 cm, soil drilling and the oven drying method were used to determine the soil moisture content at a depth of 0–50 cm. Three sets of soil samples (corresponding to P1, P2, and P3 slopes) were obtained using a cylindrical core sampler with a sampling interval of 5 cm. Meteorological factors, including photosynthetically active radiation (PAR), atmospheric temperature (T), and relative humidity (RH) were monitored by an LSI-LASTEM automatic weather station (located 30 m away from the sap flow sampling plots). The data acquisition time, which was set to 30 min, was taken simultaneously with the sap flow observations. The synergistic effect of T and RH is reflected comprehensively by the vapor pressure difference (VPD) index [27], VPD refers to the difference between the saturated vapor pressure and the actual vapor pressure in the air at a certain temperature. VPD affects stomatal closure of plants, thus controlling physiological processes such as transpiration and photosynthesis, and has an important impact on evapotranspiration and water use efficiency of forest ecosystem, which is expressed by the following equation:

$$VPD=0.611e^{\frac{17.502T}{T+240.97}}(1-RH)$$ (1)

Pei Zhiyong et al. [19] have did some researches about the sap flow of *Salix psammophila* and its response to meteorological factors, he found out that PAR, T, RH, and VPD were the main indexes, so PAR, T, RH, and VPD were selected as the meteorological factors. Guo Ying et al. [28] proposed that, because of the short duration and discontinuous character of wind speed, the relationship between sap flow rate and wind speed is rather obscure. Therefore, when selecting meteorological factors, the wind speed was not used as an analytical index. In order to verify the relationship between stem flow and environmental factors, use the formula (2) to measure the fitting effect of regression analysis model [29]:

$$AIC=n*ln(\frac{RSS}{n})+2(K+1)$$ (2)

In the formula (2), AIC indicates the Akaike Information Criterion; RSS is the sum of squared residuals; n is the sample size; K means the number of model independent variables; The smaller the AIC and RSS, the better the model fits.

### Determination of leaf area

A LAI-2200C Plant Canopy Analyzer was used to determine the Leaf Area Index of the *Salix psammophila* shrubs in the sample plots. The analyzer sensor was placed in the middle of each quadrat and each shrub, with care being made to avoid any branches that could block the field of vision of the sensor. At the same time, care was also taken to ensure that no surface vegetation or nearby shrub branches appeared in the field of vision of the canopy analyzer.

#### Data processing

Microsoft Excel 2016 was used to organize the collected data, data processing and plotting were completed with software Origin; the methods of Pearson correlation analysis and Stepwise regression analysis were used for data processing and analysis.

## Results and analysis

### Comparative analysis of *Salix psammophila* growth on different slopes

Topography and soil are important factors affecting vegetation communities, vegetation growth, and soil nutrient content change with topography [28]. Topographic factors such as slope direction, slope position, gradient, altitude, and valley width have specific functions in the ecosystem, but they are also significantly affected by the compound body of topographic factors [30]. Table 1 shows that the growth condition of *Salix psammophila* varies slightly at different slope positions. Generally speaking, the growth condition of *Salix psammophila* at P1 was better than at other slope positions. The average crown width, total number of branches, and the LAI showed a trend of P1 > P2 > P3, which means that gentle slope conditions are conducive to the growth of *Salix psammophila*.

**Table 1:** Growth status of *Salix psammophila* on different slopes.

Slope position	Elevation /m	Canopy density	Porosity /%	Average crown /cm	Average height /cm	Number of branches	Withered rate /%	*LAI*_max_
P1	1088	0.81	24.58	340 × 330	337	125	13	3.22
P2	1115	0.72	27.88	342 × 301	350	118	17	2.19
P3	1275	0.78	29.32	322×275	356	109	20	1.87

**Fig 2:**
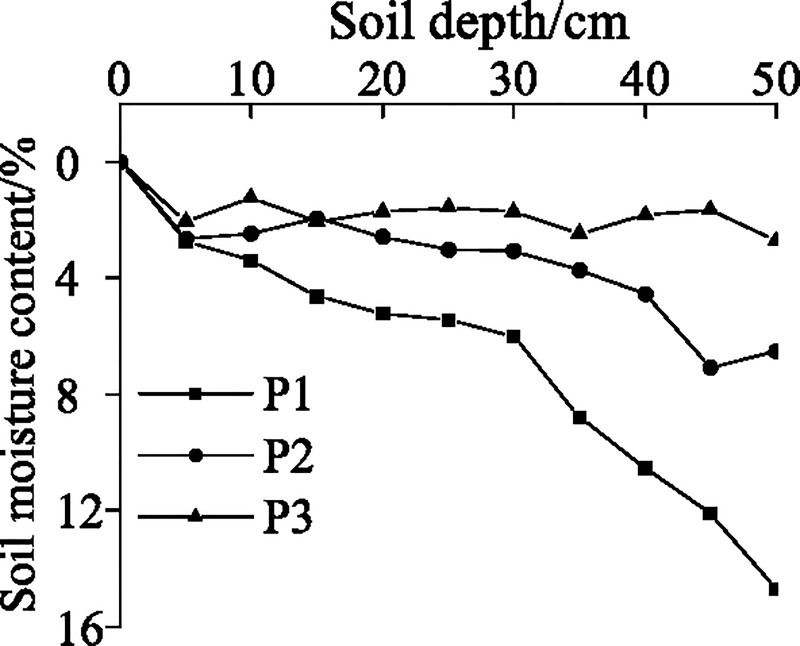
Soil water content at different slope positions.

**Fig 3:**
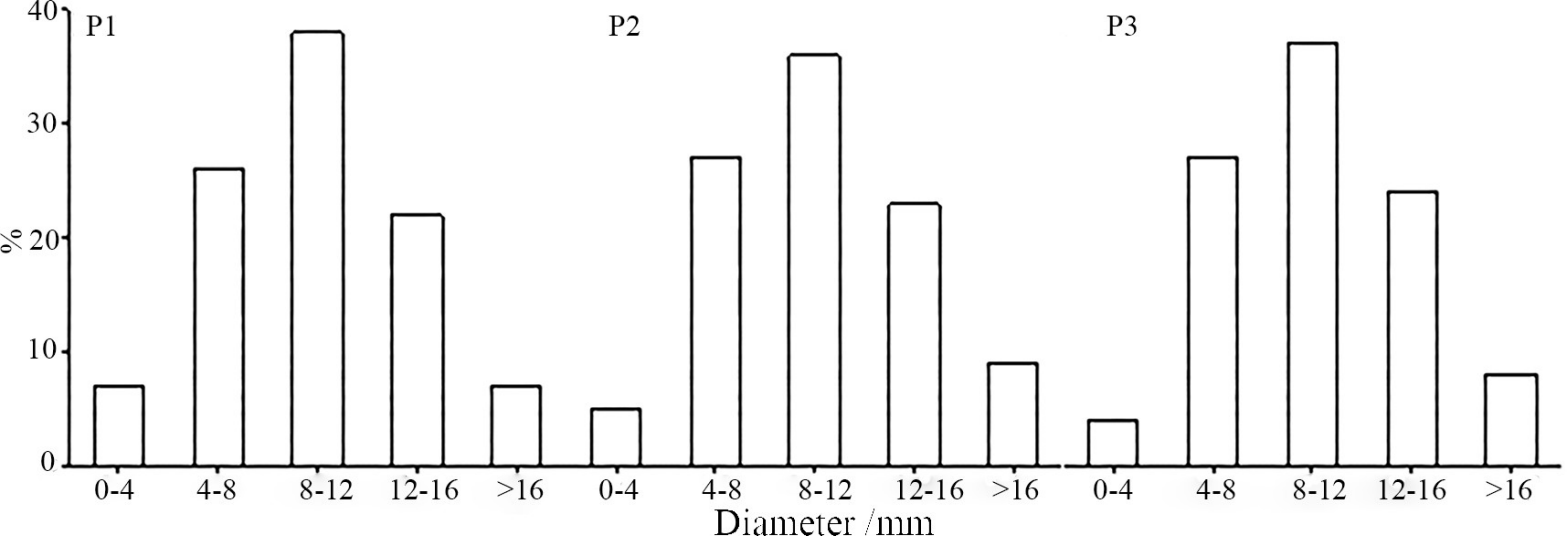
Distribution of *Salix psammophila* diameters on different slopes.

As the plants in P3 acted as a sand-fixing barrier, their average height was relatively high, indicating apical effect. The change in the slope position was associated with a change in lighting, nutrient, and soil conditions, that is, from P1 to P3, the amount of light on the slope surface increased, but the soil porosity also gradually increased, which contributed to the increased loss of water and nutrients. In addition, because of the effect of wind, the proportion of dead branches gradually increased, whereas the survival rate decreased, resulting in a sparser distribution of plants. Fig 2 shows that, as the soil depth at P1, P2, and P3 increases, the soil moisture content also increases; an obvious increasing trend can be observed at P1. As soil porosity increases gradually from P1 to P3, and because P1 is a relatively low and gentle slope, the soil water flows from P3 and P2 to P1, so that the soil moisture content assumes a trend of P1 > P2 > P3. It is evident from Fig 3 that the branch diameters of *Salix psammophila* show a normal distribution at all three slope positions, with most branches falling into the 8–12 mm range, followed by the 4–8 mm range. The low proportion of newly sprouted *Salix psammophila* branches can be attributed to nutrient loss and the high altitude.

### Changes in sap flow at different slope positions

**Fig 4:**
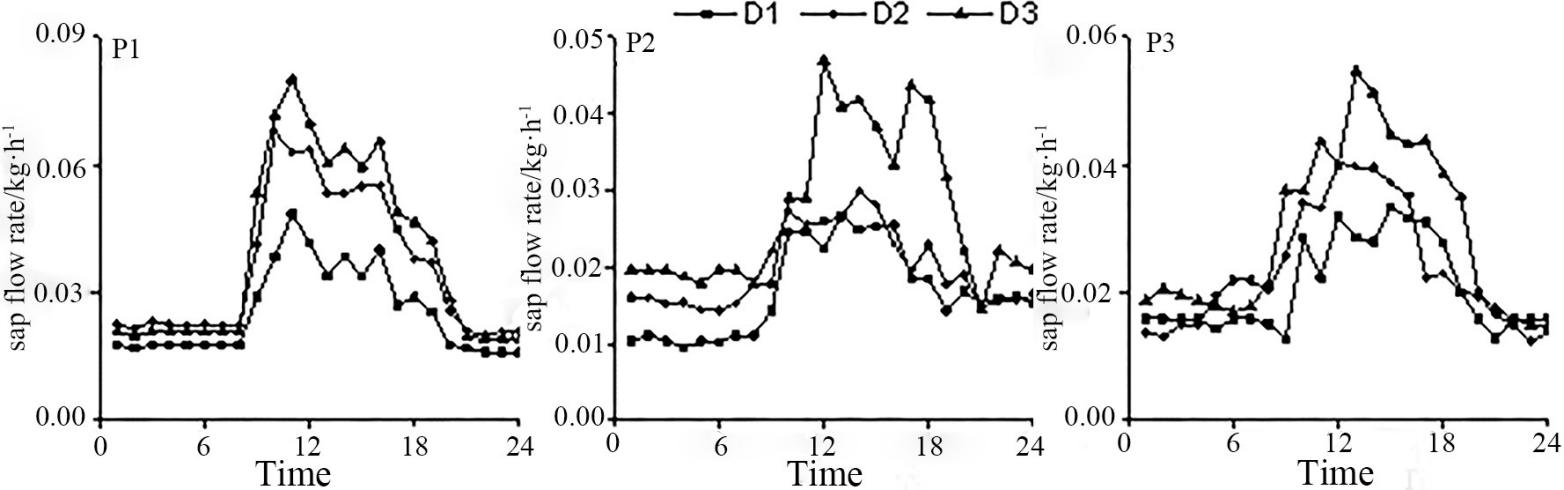
Changes in sap flow of *Salix psammophila*.

It is evident from Fig 4 that the daily variations in sap flow of *Salix psammophila* show similar trends at all slope positions, but the sap flow rates of the same slope position show slight variations because of the differences in branch diameters, with an overall trend of D3 > D2 > D1. In addition, the sap flow rate shows an obvious diurnal-nocturnal variation pattern, with a high sap flow rate during the day and a low flow rate at night, which is represented by a multi-peak bell-shaped curve. Because of the differences in altitude, the response time of *Salix psammophila* to solar radiation showed slight variations; generally, P3 specimens responded earlier than P2 specimens, and P2 specimens responded earlier than P1 specimens. The daily sap flow rate fluctuated greatly at peak value, which can be explained as follows: Strong solar radiation and high humidity at noon induce the opening of stomata, thereby increasing stomatal conductance. On the other hand, when transpiration increases, the stomata are induced to close again, thereby reducing stomatal conductance and preventing an excessive loss of water. Consequently, the sap flow peak rate shows high fluctuations, so the phenomenon of short "noon rest" from transpiration can be observed in *Salix psammophila* at different slope positions. To replenish the water consumed during diurnal transpiration and restore the water balance, the root systems of the plants change their water uptake from passive to active so that slight nocturnal sap-flow activity occurs in *Salix psammophila* at P1, P2, and P3.

**Fig 5:**
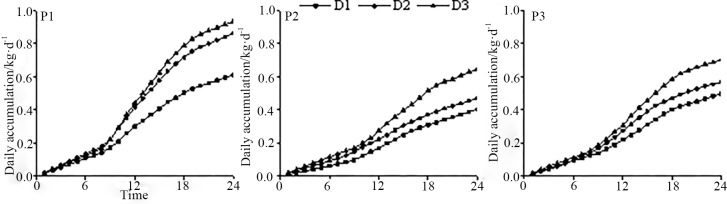
Diurnal daily accumulation changes of *Salix psammophila’*s branch.

Fig 5 shows that the trends of the diurnal accumulation of sap flow of *Salix psammophila* are consistent for all slope positions, that is, generally, the accumulation value tends to increase first and then remain stable. Concerning branch diameters of *Salix psammophila*, the daily sap flow accumulation at P1, P2, and P3 showed a trend of D3 > D2 > D1, indicating a correlation between sap flow and branch diameter. Individual *Salix psammophila* plants are fan-shaped, with most new shoots surrounded by partly mature or mature branches. As the specimen distribution at P1 was denser than that of other slope positions, the daily sap flow accumulation in D1 branches of P1 specimens was significantly different from that of D2 or D3 branches. On the other hand, because the canopy densities of P2 and P3 were lower than that of P1, with sufficient solar radiation, the differences between the sap flow accumulations of D1, D2, and D3 branches at these slope positions were not as obvious as those of P1 specimens. Variance analysis (a paired t-test) was used to analyze sap flow of *Salix psammophila* branches using the same diameter ranges. It is evident from Fig 5 and Table 2 that the cumulative daily sap flow shows a trend of P1 > P2 > P3. The results of variance analysis show that, within the same diameter range, there is no significant difference between the sap flow in D1 branches of P1 and P2 specimens, and in all three diameter ranges, there are no significant differences between P2 and P3 specimens. Based on the above results, P2 represents a transition section from P3 to P1, and P3 serves as a sand-fixing barrier, which allows the *Salix psammophila* plants at P1 to provide the maximum ecological benefit.

**Table 2:** Paired T test for the same diameter range of *Salix psammophila*’s sap flow.

Plot	Branch	P1			P2		
		D1	D2	D3	D1	D2	D3
P2	D1	0.066	-	-	-	-	-
	D2	-	0	-	-	-	-
	D3	-	-	0	-	-	-
P3	D1	0	-	-	0.596	-	-
	D2	-	0	-	-	0.124	-
	D3	-	-	0	-	-	0.318

### Relationship between *Salix psammophila* sap flow and environmental factors at different slope positions

The Pearson method was used to analyze the correlation between sap flow and meteorological factors during three consecutive typical sunny days following the full leaf development period (Table 3). The results revealed that the correlation between sap flow and various meteorological factors showed no significant differences at the P1, P2, and P3 positions, that is, all factors were significantly correlated. More specifically, RH showed a significant negative correlation, whereas the remaining factors showed a significant positive correlation, with most having a correlation value of above 0.7. Among them, PAR showed the highest correlation, followed successively by T, VPD, and RH. On the other hand, the magnitude of the correlation coefficients varied at different slope positions. For example, because of the high canopy density at P1, the correlation between sap flow and PAR showed a trend of P1 > P3 > P2; whereas T, RH, and VPD showed a trend of P3 > P1 > P2.

**Table 3:** Correlation Analysis between sap flow rate and environmental factors on different slopes.

Plot	Branch	PAR	T	RH	VPD
P1	D1	0.807**	0.720**	-0.490**	0.670**
	D2	0.820**	0.742**	-0.509**	0.693**
	D3	0.810**	0.742**	-0.510**	0.687**
P2	D1	0.464**	0.357**	-0.246**	0.370**
	D2	0.822**	0.711**	-0.583**	0.682**
	D3	0.726**	0.735**	-0.574**	0.721**
P3	D1	0.727**	0.726**	-0.552**	0.720**
	D2	0.848**	0.695**	-0.457**	0.660**
	D3	0.818**	0.808**	-0.621**	0.780**

Note

“**”indicates that the correlation is significant at the 0.01 level

The results of a correlation analysis reveal that the sap flow rate of *Salix psammophila* at different slope positions is closely related to meteorological factors. Therefore, to further characterize the relationship between the sap flow rate at different slope positions and environmental factors, this study used the multiple linear regression method. The stepwise regression method was used to gradually eliminate factors that had a small influence on the sap flow rate and to obtain the optimal regression model (Table 4). It is evident from Table 4 that all the regression coefficients reached a level of high significance, with most R^2^ values being above 0.7, which indicates that the response of the sap flow rate to environmental factors can be expressed well by the regression equation. Also, the value of RSS and ACI are relatively small, which indicate the stepwise regression model has good goodness of fit. Concerning the selected variables, there were slight differences between P1, P2, and P3, but at all three slope positions, PAR and T still had a dominant effect and showed a positive correlation. At P1, VPD was negatively correlated with the sap flow rate of D1, D2, and D3 branches, whereas RH was only correlated with the sap flow rate of D3, with the two factors being negatively correlated. At P2, only PAR and T were selected for D1 and D3, whereas the sap flow of D2 was affected by all four meteorological factors, with RH and VPD also showing a negative correlation. At P3, PAR and T were selected for D1, D2, and D3, with all of them being positively correlated, indicating that the upper slope vegetation is highly sensitive to these two factors.

**Table 4:** Stepwise Regression analysis of sap flow rate and environmental factors of *Salix psammophila* on different slopes.

Plot	Branch	Selected variable					*R*^2^	*F*	*P*	*RSS*	*AIC*
		PAR	T	RH	VPD	Intercept					
P1	D1	0.681	0.719	-	-0.530	0.010	0.711	56.668	0.000	0.002	-217.42
	D2	0.671	0.723	-	-0.504	0.09	0.738	64.628	0.000	0.005	-195.43
	D3	0.717	1.131	-1.258	-0.371	0.028	0.745	49.708	0.000	0.008	-182.15
P2	D1	0.464	-	-	-	-0.010	0.215	19.457	0.000	0.044	-147.24
	D2	0.849	1.341	-0.850	-1.981	0.028	0.797	66.755	0.000	0.001	-232.06
	D3	0.402	0.437	-	-	0.011	0.614	55.583	0.000	0.003	-209.69
P3	D1	0.420	0.416	-	-	0.008	0.607	54.094	0.000	0.002	-219.42
	D2	0.848	-	-	-	0.019	0.719	181.418	0.000	0.002	-221.42
	D3	0.486	0.449	-	-	0.006	0.760	110.585	0.000	0.003	-209.69

Note: *R*^2^ indicates Determination coefficient or goodness of fit, it is used to measure the degree to which the estimated model fits the observed value; *F* value is the result of Analysis of variance, which is an overall test of the whole regression equation. It means whether the whole regression equation has any use value. If the *P* value corresponding to *F* value is less than 0.05, the regression equation can be considered useful.

## Discussion

In this study, the sap flow rates at P1, P2, and P3 showed obvious daily variations expressed graphically by a multi-peak bell-shaped curve. These results are consistent with those of Pei Zhiyong et al. [19] and Zhang Jing et al. [31], who studied the sap flow rates of *Salix psammophila* and apple trees, respectively. This result revealed that the transpiration patterns of psammophytes are similar to that of other vegetation such as trees. The results of this study reported that the daily sap flow accumulation of *Salix psammophila* at different slope positions followed a pattern of D3 > D2 > D1 and had an overall trend of P1 > P3 > P2. The results of Wang Qiangmin et al. [18] also came up with that the sap flow rate of *Salix psammophila* changed for different branch diameters, indicating a positive correlation between the sap flow rates and branch diameters. Wang Yanbing et al. [32] found that the daily mean sap flow rate of *Larix principis-rupprechti* at different slope positions in a semi-arid region showed a trend of lower slope > upper position > middle slope, which is consistent with the results of the present study. Similarly, by setting up five plantation plots located at the upper, middle-upper, middle, middle-lower, and lower slope positions (P1, P2, P3, P4, and P5) in one of the watersheds of the Liupan Mountains, Wang Yunni et al. [33] found that there were differences in the sap flow rates of *Larix principis-rupprechtii* at different slope positions, with a trend of P2 > P4 > P3 > P1 > P5. The reason is that soils in lower slope positions in sagebrush ecosystems, for example, have greater soil oisture, higher soil organic matter content, and higher rates of nitrogen mineralization and gaseous losses than do upslope soils. Therefore, it can be concluded that there are similarities between the sap flow rates of desert shrubs and those of other vegetation under the same meteorological conditions, whereas the sap flow rates of the same type of vegetation but at different slope positions and with different environmental conditions show some variations. This means that terrain and vegetation growth are closely related.

A large number of studies have been done on the relationship between sap flow and meteorological factors [34–36], but the obtained results are not completely consistent, as many factors can drive transpiration, which indicates that the dominant factors might vary. For example, Huang Lei et al. [23] and Han Lei et al. [37,38] respectively studied the sap flow rates of *Artemisia ordosica* and *Caragana korshinskii*, they found that photosynthetically active radiation was the primary environmental factor affecting sap flow; Guo Yue et al.[39] found that the sap flow rate of *Hedysarum scoparium* in the Mu Us Desert was affected primarily by photosynthetically active radiation and wind speed; and Zhang Jing et al. [31] found that only soil moisture and vapor pressure deficit were significantly correlated with the sap flow velocity of apple trees. It is evident that the sensitivity of desert shrubs and trees to environmental factors varies because of the differences in climate. The results of a Pearson correlation analysis showed that there was no correlation between the sap flow of *Salix psammophila* and meteorological factors at different slope positions; however, a significant positive correlation was found with PAR, T, and VPD, and a significant negative correlation was found with RH, which is consistent with the results of previous studies [40, 41]. It was also found that *Salix psammophila* at P1, P2, and P3 had different response times to meteorological factors. This was attributed to the varying sunlight-projection angle at different slope positions, which affected the amount of solar radiation at each slope position, subsequently affecting other ecological factors, such as atmospheric temperature. After further analysis using the stepwise regression model, it was found that PAR and T had a dominant effect on the sap flow of *Salix psammophila* at different slope positions. In addition, the P1 specimens were also significantly affected by VPD. The main reason is that the diurnal and climatic differences in air temperature and humidity determine the driving force for transpiration. Air inside the leaf is always saturated with water vapor because it is adjacent to moist cell surfaces. On sunny day, air temperature rises to a maximum shortly after midday, allowing the air to hold more water. This rise in air temperature and the radiation absorbed by the leaf increases the temperature of the leaf and therefore the water vapor concentration inside the leaf. The water vapor concentration of the external air generally increases less than that inside the leaf. The resulting increase in the gradient in water vapor concentration between the inside and the outside of the leaf increases the transpirational water loss from the leaf. In evening the temperature decreases, causing a decline in the water vapor concentration inside the leaf and a decline in transpiration. In this study, we analyzed the daily dynamic changes of *Salix psammophila* sap flow and its relationship with meteorological factors at different slope positions under typical sunny conditions. Further studies are needed to determine, on a long-term scale, whether sap flow rates at different slope positions vary under sufficient-water-supply and drought-stress conditions, which is essential for a more accurate estimation of water consumption through transpiration of *Salix psammophila*.

## Conclusions

(1) Among the three different slopes, P1 had the highest moisture content and the healthiest specimens of *Salix psammophila*, followed by P2. P3 had the highest proportion of dead branches and a low survival rate, but with obvious apical dominance, while P1 has the most suitable environment for the growth of *Salix psammophila*.

(2) The daily variation patterns of sap flow of *Salix psammophila* at the three slope positions were basically the same: each pattern could be represented by a multi-peak bell-shaped curve, with minute fluctuations in sap flow during the nighttime. The cumulative daily flow of *Salix psammophila* showed a variation trend of P1 > P3 > P2, with P1 exhibiting significantly different values from P2 and P3, whereas the values for P2 and P3 were not significantly different.

(3) The sap flow of *Salix psammophila* at all three slope positions was significantly correlated with meteorological factors. Among them, PAR, T, and VPD were significantly and positively correlated, and RH was significantly and negatively correlated. Furthermore, in the multiple linear regression model, the meteorological factors selected for different slope positions and branch diameters varied slightly, but RA and T had a dominant effect at all slopes and diameters.

## Supporting information

S1 File Dataset. (XLSX)
